# Another polymorphic mitochondrial genome of *Grampus griseus* and phylogeny of family Delphinidae

**DOI:** 10.1080/23802359.2021.1959453

**Published:** 2021-08-06

**Authors:** Jayan Duminda M. Senevirathna, Ryo Yonezawa, Taiki Saka, Yoji Igarashi, Kazutoshi Yoshitake, Shigeharu Kinoshita, Noriko Funasaka, Shuichi Asakawa

**Affiliations:** a Laboratory of Aquatic Molecular Biology and Biotechnology, Department of Aquatic Bioscience, Graduate School of Agricultural and Life Sciences, The University of Tokyo, Tokyo, Japan; b Department of Animal Science, Faculty of Animal Science and Export Agriculture, Uva Wellassa University, Badulla, Sri Lanka; c Department of Life Sciences and Chemistry, Graduate School of Bioresources, Mie University, Tsu, Japan; d Department of Life Sciences, Graduate School of Bioresources, Mie University, Tsu, Japan

**Keywords:** Mitogenome, polymorphism, phylogeny, dolphins

## Abstract

Risso’s dolphin (*Grampus griseus* Cuvier, 1812) is the only species of genus *Grampus* and a cosmopolitan marine inhabitant. Here, we report a polymorphic complete mitochondrial genome of *G. griseus*. The size of the total mitochondrial genome was 16,386 bp in length and contains 13 protein-coding genes, 22 transfer RNA genes, two ribosomal RNA genes, and a control region. 37 single nucleotide polymorphic sites (SNPs) were identified compared to the references. Based on the available total mitochondrial dolphin genomes’ phylogenetics, *G. griseus* has formed a clade with 0.1415 distance, sister to the following species of the subfamily Globicephalinae and the taxonomy of *Orcinus orca* still needs further investigations.

Significant features of Risso’s dolphins are the tallest dorsal fin compared to the body size in any cetaceans, unusual dentition, and specific skin morphology in adults (Baird 2009). Based on mitochondrial genome analysis, the Risso’s dolphin has clustered away from other Delphinidae species (Xiong et al. 2009). After that, the phylogenetic status of *G. griseus* has confirmed under the subfamily Globicephalinae species (Cunha et al. 2011). There are limited public genomics data on Risso’s dolphin. Therefore, the mitochondrial genome of *G. griseus* was sequenced, assembled, annotated, and its polymorphic characteristics were explained.

A muscle tissue sample of *G. griseus* (sample ID; 19TK411) was received from fishermen of the Taiji Fishery Association under the cooperation of the biological surveys by the National Research Institute of Far Seas Fisheries, Japan Fisheries Research and Education Agency. The voucher sample was stored as UMUT RV20210330-01 at The University Museum (http://www.um.u-tokyo.ac.jp/hp/sasaki/index.htm; web-master@um.u-tokyo.ac.jp), The University of Tokyo. Total genomic DNA was extracted using a DNA Isolation Kit (Qiagen, Hilden, Germany). DNA library was prepared by Nextera DNA Flex library prep kit (Illumina, San Diego, CA, USA), and sequencing was performed by the Illumina HiseqX next-generation sequencing platform. The sequence data were processed and mapped to the reference mitochondrial genome (NC 012062.1) by the CLC Genomic Workbench ver.8.0.1 (Qiagen). The CLC produced consensus sequence was processed into a featured mitochondrial genome using the annotation function of the Geneious ver.8 (Tomy Digital Biology CO., LTD, Tokyo, Japan) by annotating to a GenBank format data file of the reference (Supplementary Figure 1). The final mitogenome of our sample was submitted to the DNA Data Bank Japan (DDBJ) and accession number (LC630882) was obtained. The complete mitogenomes of the family Delphinidae were collected from NCBI GenBank. Global alignment and phylogenetic analysis of the mitogenomes was performed using the Tamura-Nei model (Tamura and Nei 1993), and Neighbor-Joining (NJ) method (Saitou and Nei 1987) with an out-group of family Physeteridae by Tree Builder of Geneious ver.8 (Supplementary Figure 2). A maximum likelihood tree was also generated by MEGA X (Kumar et al. 2018) to further confirm the phylogeny of the family Delphinidae (Figure 1).

**Figure 1.**
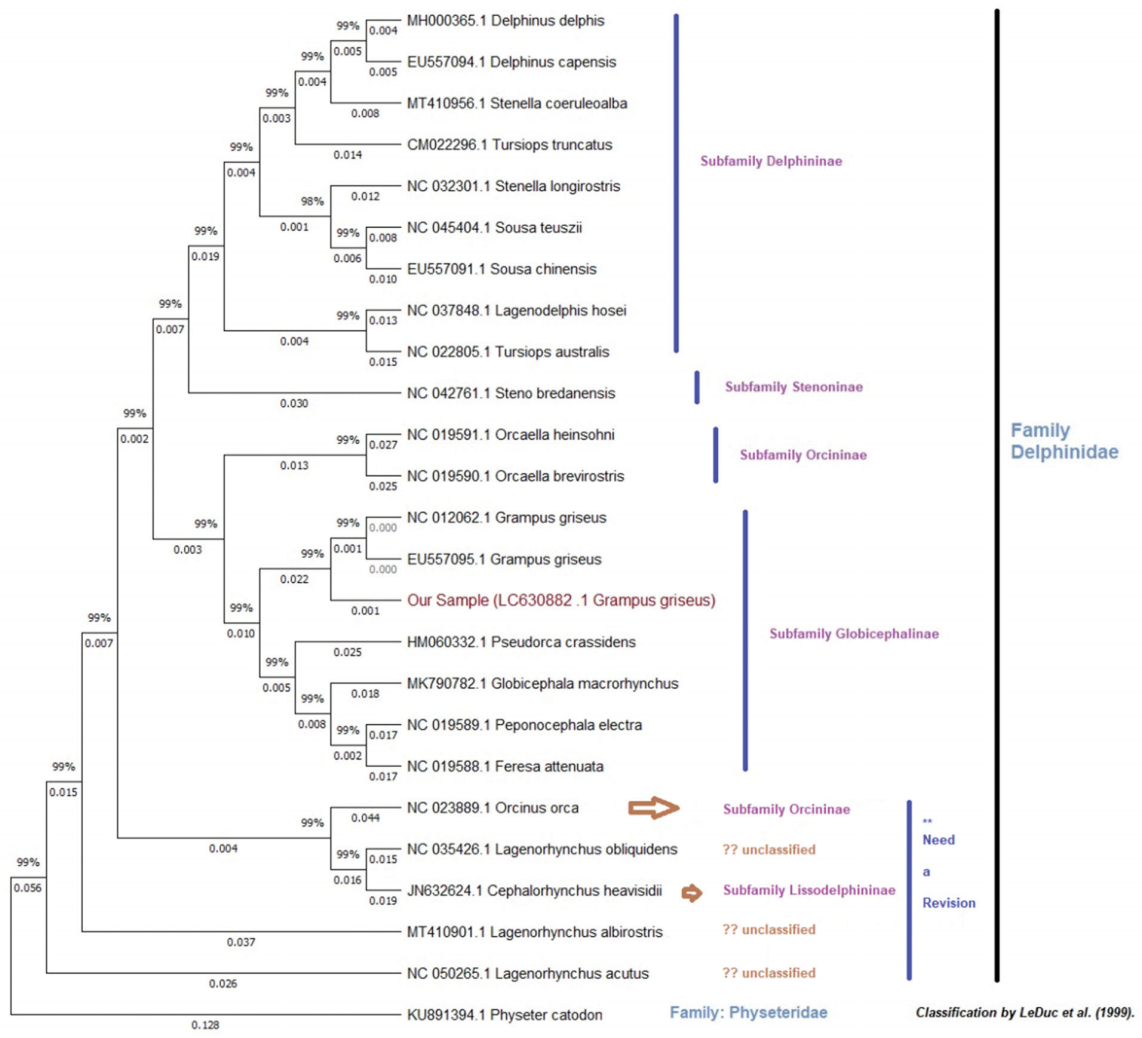
The maximum likelihood tree of *Grampus griseus* of this study and additional 24 species of family Delphinidae performed using total mitogenomes of around 16,000 bp. The bootstrap support values are indicated on each node. The clades were identified according to the recent classification of family Delphinidae by LeDuc et al.

Our sample’s mitogenome of *G. griseus* was 16,386 bp in length and contained a set of 13 protein-coding genes (PCGs), 22 transfer RNA (tRNA) genes, two ribosomal RNA (rRNA) genes, and one non-coding control region (Supplementary Figure 1). The overall nucleotide composition was 33% A, 26.3% C, 12.8% G, and 27.9% T, indicating contents of AT (60.9%), and GC (39.11%). Among the 13 PCGs, three start codons were found: ‘ATA’ (at ND3, ND5), ‘ATT’ (at ND2), and others were ‘ATG.’ Seven PCGs had a ‘TAA’ stop codon; however, ND2, COI, COIII, ND3, ND4, and CYTB did not. The gene arrangement of *G. griseus* was identical to that of other species of the family Delphinidae published in the NCBI GenBank database. Estimation of evolutionary divergence identified 0.0023 pairwise distance to the references (NC 012062.1, and EU557095.1). We recognized 37 single nucleotide polymorphism (SNP) sites between our sample, and the references, indicating a potentially high level of intraspecific variation. As a summary, SNPs were two at 16s rRNA, three at ND1, five at ND2, two at COX1, two at COX2, one at ATP8, one at ATP6, two at ND4L, one at ND4, seven at ND5, four at CYTB, and seven at D-loop regions (Supplementary Tables 1 and 2). The reference mitogenomes, NC 012062.1, and EU557095.1 were identical. Identification of these variable sites will be important to develop biomarkers for various studies, like population genomics.

The phylogenetic tree showed that a clade of the subfamily Globicephalinae, with *G. griseus*, distinctly formed a monophyletic clade with subfamily Orcininae. Also, *G. griseus* shows 0.1415 distance to the other species of the same subfamily and it was supported by previous studies (Leduc et al. 1999; Cunha et al. 2011). The subfamilies of Delphininae and Stenoninae are grouped into a single monophyletic clade (Figure 1). *Orcinus orca* has grouped with subfamily Lissodelphininae with supporting (Horreo 2019) and contradicting (Lee et al. 2018) that shows potential reinvestigations of the taxonomy (Figure 1 and Supplementary Figure 2).

## Data Availability

The genome sequence data that supported the findings of this study are openly available in GenBank of DDBJ/NCBI at [https://www.ddbj.nig.ac.jp/index-e.html] (https://www.ncbi.nlm.nih.gov/) under the accession no. LC630882. The associated BioProject, DRA/SRA, and Bio-Sample numbers are PRJDB11931, DRA012378, and SAMD00389444 respectively.
